# Clinical advantages of two vs. three courses of neoadjuvant chemotherapy using docetaxel + cisplatin + 5-fluorouracil to improve preoperative nutritional status and mitigate decreasing skeletal muscle in resectable esophageal cancer

**DOI:** 10.1007/s10147-025-02839-6

**Published:** 2025-07-17

**Authors:** Kazuaki Matsui, Yutaka Miyawaki, Ryota Kobayashi, Masatoshi Yoshizawa, Tetsuro Toriumi, Gen Ebara, Hiroshi Sato, Shinichi Sakuramoto

**Affiliations:** Department of Gastroenterological Surgery, Saitama Medical University International Medical Center, Hidaka, Saitama 1397-1, Yamane350-1298 Japan

**Keywords:** Esophagectomy, Nutrition, Skeletal muscle

## Abstract

**Purpose:**

Neoadjuvant chemotherapy (NAC) using docetaxel/cisplatin/5-fluorouracil (DCF) for locally advanced esophageal cancer (EC) showed better clinical outcomes than conventional regimens; however, had high incidence of serious adverse events.

**Methods:**

Patients who underwent radical esophagectomy after neoadjuvant-DCF were classified into two-course and three-course groups (*n* = 60 and 41). Multiple clinical indicators related to nutrition and skeletal muscle that were reported to be associated with survival outcomes were compared between the two groups.

**Results:**

Changes in prognostic nutritional index (PNI), geriatric nutritional risk index (GNRI), and psoas muscle area (PMA) were significantly low in the three-course group (*p* < 0.001, < 0.001, and = 0.003). Multivariate analyses for PNI change rate showed initial PNI < 45 and three-course DCF as independent associated factors (*B* = 0.129; *p* < 0.001 and *B* =  − 0.057; *p* = 0.022); GNRI change rate showed body mass index ≥ 21, initial PNI < 45, and three-course DCF as independent associated factors (B =  − 0.033; *p* < 0.001, *B* = 0.062; *p* < 0.001, and *B* =  − 0.059; *p* < 0.001); PMA change rate showed three-course DCF and cStage IV as independent associated factors (*B* =  − 0.024; *p* = 0.011 and *B* =  − 0.025; *p* = 0.038). There were not significant differences in the long-term survivals between the two groups in pStages I–IV.

**Conclusions:**

Two courses were superior to three courses for improving nutritional status and mitigating skeletal muscle decreasing during NAC–DCF for EC.

**Supplementary Information:**

The online version contains supplementary material available at 10.1007/s10147-025-02839-6.

## Introduction

Although multidisciplinary treatments for advanced esophageal cancer (EC) have been improved, long-term survival after esophagectomy for EC is worse than for other digestive cancers [1]. EC can show widespread metastasis even in the early stage [2, 3] and early recurrence even after highly invasive radical esophagectomy [4, 5]. In Japan, the combination of neoadjuvant chemotherapy (NAC) followed by radical surgery is the primary standard treatment for advanced EC. The Japanese Clinical Oncology Group (JCOG) 1109 study showed that oncological efficacy was better for the neoadjuvant docetaxel + cisplatin + 5-fluorouracil (DCF) regimen than for the previous standard regimen with cisplatin + 5-fluorourail (CF); however, the toxicity was worse for NAC using DCF than for NAC using CF [6]. The JCOG 1109 study showed that NAC using the DCF regimen followed by radical surgery should be the standard treatment for locally advanced EC [7].

Increasing the intensity of the NAC regimen from CF to DCF improved treatment outcomes, but the incidence of serious adverse events, including leukocytopenia, during NAC was significantly increased [6]. A strong triplet regimen can still be problematic for some groups, such as elderly patients, those with severe malnutrition, and those with various comorbidities. In the JCOG 1109 study, the neoadjuvant DCF group received three courses of docetaxel (70 mg/m^2^), cisplatin (70 mg/m^2^), and 5-fluorouracil (750 mg/m^2^) repeated every 3 weeks. Meanwhile, Makino et al. and Shiraishi et al. reported a trial that compared two vs. three courses of neoadjuvant DCF treatment for locally advanced esophageal squamous cell carcinoma, and they concluded that a two-course DCF regimen had potential as an optional NAC treatment [8, 9]. These studies demonstrated that three courses compared with two courses of DCF provided a better NAC response without increasing the incidence of adverse events, whereas there were no significant differences in the overall and progression-free survival rates between the two- and three-course DCF groups.

Still, the number of DCF courses as a NAC regimen might not yet be unified by all physicians. Especially in the patients with poor general conditions, two courses of DCF instead of three potentially can be an acceptable neoadjuvant treatment. This retrospective study aimed to assess the difference in influence on nutritional status and skeletal muscle loss between two and three courses of neoadjuvant DCF as a NAC regimen.

## Patients and methods

### Patients

This study retrospectively reviewed 106 patients who underwent esophagectomy after two or three courses of DCF as a NAC regimen for locally advanced thoracic EC at Saitama Medical University International Medical Center between May 2017 and May 2024. Patients with EC having invasion to cervical esophagus as well as patients who received NAC at other institutions were excluded. EC with cervical invasion was excluded, because the treatment strategy for cervical EC generally includes radiotherapy. The analyzed patients were classified into two-course and three-course DCF groups. Preoperative changes in nutritional status and skeletal muscle volume as well as long-term survival were compared between the two groups. Furthermore, to clarify the effect of the difference in the number of NAC courses on the elderly population, these preoperative indicators were investigated in the patients who were ≥ 75 years.

### Neoadjuvant chemotherapy

One DCF regimen cycle comprised docetaxel (70 mg/m^2^), cisplatin (70 mg/m^2^), and 5-fluorouracil (750 mg/m^2^) and was repeated every 3 weeks. Regarding the choice of two or three courses of DCF, although three courses were administered as a standard regimen in the early period of this study (2017–2021), two courses were used as the standard regimen in the later period (2022–2024) for decreasing severe adverse events during neoadjuvant treatment at our institution; however, in several patients, such as those with cStage IV EC, two courses plus one additional course were administered on the basis of a clinical decision even in the later period. The standard treatments to prevent adverse events were quinolone antibiotics from days 5 to 15 and granulocyte colony-stimulating factor 24 h after the end of continuous administration of 5-fluorouracil. Regarding adverse events, given the retrospective design of the study, hematological adverse events that could be objectively evaluated from blood-test results were evaluated according to the Common Terminology Criteria for Adverse Events (CTCAE) grading [10].

### Evaluation of nutritional status

Regarding nutritional status, the prognostic nutritional index (PNI, 10 × albumin [g/dl] + 0.005 × total lymphocytes count [/mm^3^])[11], geriatric nutritional risk index (GNRI, 14.89 × albumin [g/dl] + 41.7 × body weight [kg]/ideal body weight [kg]) [12], neutrophil-to-lymphocyte ratio (NLR) [13], and Glasgow prognostic score (GPS, score = 0; C-reactive protein [CRP] < 1.0 mg/dl and albumin ≥ 3.5 g/dl, score = 1; CRP ≥ 1.0 mg/dl or albumin < 3.5 g/dl, and score = 2; CRP ≥ 1.0 mg/dl and albumin < 3.5 g/dl) [14] were evaluated at two points before and after two/three courses of neoadjuvant DCF. Because this was a retrospective study, considering the initial variability in the patients’ characteristics among the two groups, the rates of changes in these indicators from the initial value to the preoperative value were evaluated in this study.

### Evaluation of skeletal muscle volume

Regarding the skeletal muscle volume, the PMA at the third lumbar vertebra (L3), that was used as one of the evaluation measures for sarcopenia, was evaluated in this study [15]. For the same reason as used in the evaluation of nutritional status, the rate of change from the initial value to preoperative value was chosen to be evaluated. In addition, the psoas muscle index (PMI, PMA [cm^2^]/square of height [m^2^]) was used to evaluate sarcopenia. In this study, sarcopenia was defined as a PMI < 6.36 for males and < 3.92 for females, as in previous studies [16, 17].

### Statistical analysis

The Chi-square and Fisher’s exact tests were used to analyze categorical variables, and the Wilcoxon rank-sum test was used for continuous variables. Univariate and multivariate analyses were performed to investigate the effect of two courses vs. three courses of DCF on each index. The Kaplan–Meier method and the log-rank test were used to generate overall survival (OS) curves and recurrence-free survival (RFS) curves. Variables with values of *p* < 0.10 in the univariate analysis were included as covariates in the multivariate analyses. All differences were considered statistically significant for values of *p* < 0.05. EZR, a modified version of R commander designed to add statistical functions, was used to perform all statistical analyses.

## Results

### Patients’ characteristics

**Fig. 1:**
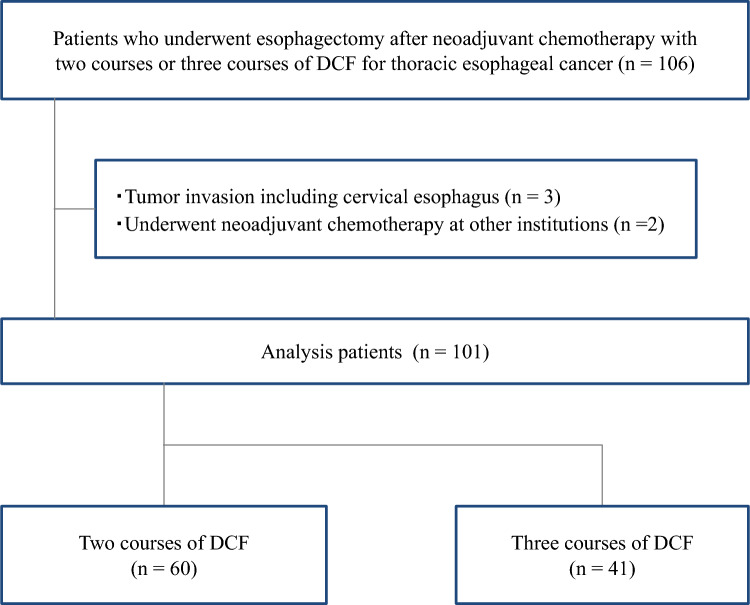
Flowchart of patient inclusion. A total of 101/106 patients, excluding six with exclusion criteria, were enrolled in the analysis. The numbers of patients who received two and three courses of docetaxel, cisplatin, and 5-fluorouracil (DCF) regimen were 60 and 41, respectively

One hundred and six patients who underwent esophagectomy after two or three courses of neoadjuvant DCF were retrospectively reviewed. Among them, three patients who had tumor invasion of the cervical esophagus and two who had received NAC at other institutions were excluded from the analysis. The analyzed patients were classified into two groups: two courses of DCF (*n* = 60) and three courses of DCF (*n* = 41) (Fig. 1). The patients’ characteristics are summarized in Table 1. The overall cohort of patients comprised 92 males and 9 females, with a mean age of 70.5 years (interquartile range [IQR]: 66.8–74.4). The mean values of the initial body mass index (BMI), initial PNI, initial GNRI, and initial NLR were 21.0 (IQR: 19.4–22.9), 47.1 (IQR: 43.7–50.3), 99.1 (IQR: 91.8–104.7), and 3.3 (IQR: 2.2–4.0), respectively. The numbers of patients with GPS 0, 1, and 2 were 79 (78.2%), 13 (12.0%), and 9 (8.9%), respectively. Among these indicators, only the initial PNI was significantly lower in the two-course group. The mean initial PMA was 15.5 (IQR: 12.9–18.1), and 64 patients (63.4%) had sarcopenia according to the PMI.

**Table 1:** Patients’ characteristics

	Total (*n* = 101)	Two courses (*n* = 60)	Three courses (*n* = 41)	*p* value
Age (years, median [IQR])	70.5 (66.8–74.4)	71.0 (67.0–74.5)	69.7 (66.8–73.3)	0.424
Sex				
Male	92 (91.1%)	56 (93.3%)	36 (87.8%)	0.480
Female	9 (8.9%)	4 (6.7%)	5 (12.2%)	
Initial body weight (kg, median [IQR])	57.5 (51.5–63.3)	58.6 (51.7–64.8)	54.8 (50.9–60.8)	0.202
Initial BMI (kg/m^2^, median [IQR])	21.0 (19.4–22.9)	21.0 (19.5–23.2)	20.9 (19.0–22.7)	0.552
Initial PNI	47.1 (43.7–50.3)	45.9 (43.1–49.4)	48.4 (46.0–51.6)	0.021
Initial GNRI	99.1 (91.8–104.7)	95.3 (91.6–104.6)	100.2 (92.3–104.6)	0.455
Initial NLR	3.3 (2.2–4.0)	3.3 (2.0–3.8)	3.3 (2.3–4.3)	0.321
Initial GPS				
0	79 (78.2%)	46 (76.7%)	33 (80.5%)	0.765
1	13 (12.9%)	9 (15.0%)	4 (9.8%)	
2	9 (8.9%)	5 (8.3%)	4 (9.8%)	
Initial PMA (cm^2^, median [IQR])	15.5 (12.9–18.1)	15.8 (13.4–18.6)	15 (12.3–17.6)	0.428
Initial sarcopenia based on PMI	64 (63.4%)	36 (60.0%)	28 (68.3%)	0.411
Tumor location				
Ut	13 (12.9%)	6 (10.0%)	7 (17.1%)	0.519
Mt	41 (40.6%)	24 (40.0%)	17 (41.5%)	
Lt	47 (46.5%)	30 (50.05)	17 (41.5%)	
Histological type				
Squamous cell carcinoma	97 (96.0%)	58 (96.7%)	39 (95.1%)	1.000
Others	4 (4.0%)	2 (3.3%)	2 (4.9%)	
cStage				
0	0 (0.0%)	0 (0.0%)	0 (0.0%)	0.001
I	1 (1.0%)	1 (1.7%)	0 (0.0%)	
II	20 (19.8%)	15 (25.0%)	5 (12.2%)	
III	61 (60.4%)	40 (66.7%)	21 (51.2%)	
IV	19 (18.8%)	4 (6.7%)	15 (36.6%)	
pStage				
0	10 (9.9%)	5 (8.3%)	5 (12.2%)	0.811
I	9 (8.9%)	6 (10.0%)	3 (7.3%)	
II	27 (26.7%)	14 (23.3%)	13 (31.7%)	
III	35 (34.7%)	22 (36.7%)	13 (31.7%)	
IV	20 (19.8%)	13 (21.7%)	7 (17.1%)	
Pathological response to NAC				
Grade 0	5 (5.0%)	4 (6.7%)	1 (2.4%)	0.252
Grade 1a	33 (32.7%)	16 (26.7%)	17 (41.5%)	
Grade 1b	26 (25.7%)	19 (31.7%)	7 (17.1%)	
Grade 2	26 (25.7%)	16 (26.7%)	10 (24.4%)	
Grade 3	11 (10.9%)	5 (8.3%)	6 (14.6%)	
Overall hematological adverse events (CTCAE grade ≥ 2)	77 (76.2%)	45 (75.0%)	32 (78.0%)	0.814
Leukocytopenia (CTCAE grade ≥ 2)	61 (60.4%)	34 (56.7%)	27 (65.9%)	0.411
Neutropenia (CTCAE grade ≥ 2)	35 (34.7%)	32 (53.3%)	31 (75.6%)	0.036
Febrile neutropenia (CTCAE grade ≥ 3)	17 (16.8%)	10 (16.7%)	7 (17.1%)	1
Anemia (CTCAE grade ≥ 2)	30 (29.7%)	15 (25.0%)	15 (36.6%)	0.269
Thrombocytopenia (CTCAE grade ≥ 2)	2 (2.0%)	0 (0.0%)	2 (4.9%)	–

*IQR* interquartile range, *BMI* body mass index, *PNI* prognostic nutritional index, *GNRI* geriatric nutritional risk index, *NLR* neutrophil-to-lymphocyte ratio, *GPS* Glasgow prognostic score, *PMA* psoas muscle area, *PMI* psoas muscle index, *NAC* neoadjuvant chemotherapy, *CTCAE* Common Terminology Criteria for Adverse Events

Regarding cancer stages, although the number of patients with cStage IV was significantly higher in the three-course group, the difference in pStage between the two groups was not significant. Although the number of patients with a grade-3 pathological response to NAC was higher in the three-course group, the difference was not significant. There were no significant differences in the overall hematological adverse events between the two groups; however, the incidence of neutropenia with CTCAE grade ≥ 2 was significantly higher in the three-course group than in the two-course group. Regarding surgical outcomes, the number of complications with CD grade ≥ 3 were higher in the three-course group, but the difference was not significant (Table 2).

**Table 2:** Surgical procedures and outcomes

	Total (*n* = 101)	Two courses (*n* = 60)	Three courses (*n* = 41)	*p* value
Surgical approach				–
Open thoracotomy	3 (3.0%)	0 (0.0%)	3 (7.3%)	
Thoracoscopic/robot-assisted surgery	98 (97.0%)	60 (100.0%)	38 (92.7%)	
Operative time (min, median [IQR])	432 (388–456)	435 (393–450)	426 (386–472)	0.945
Blood loss (ml, median [IQR])	195 (108–330)	202 (112–288)	167 (94–365)	0.693
All complications with CD grade ≥ 3	18 (17.8%)	7 (11.7%)	11 (26.8%)	0.065
Pneumonia	12 (11.9%)	7 (11.7%)	5 (12.2%)	1.000
Anastomotic leakage	11 (10.9%)	7 (11.7%)	4 (9.8%)	1.000

*IQR* interquartile range, *CD* Clavien–Dindo

### Comparisons of changes in nutritional status and skeletal muscle volume

**Fig. 2:**
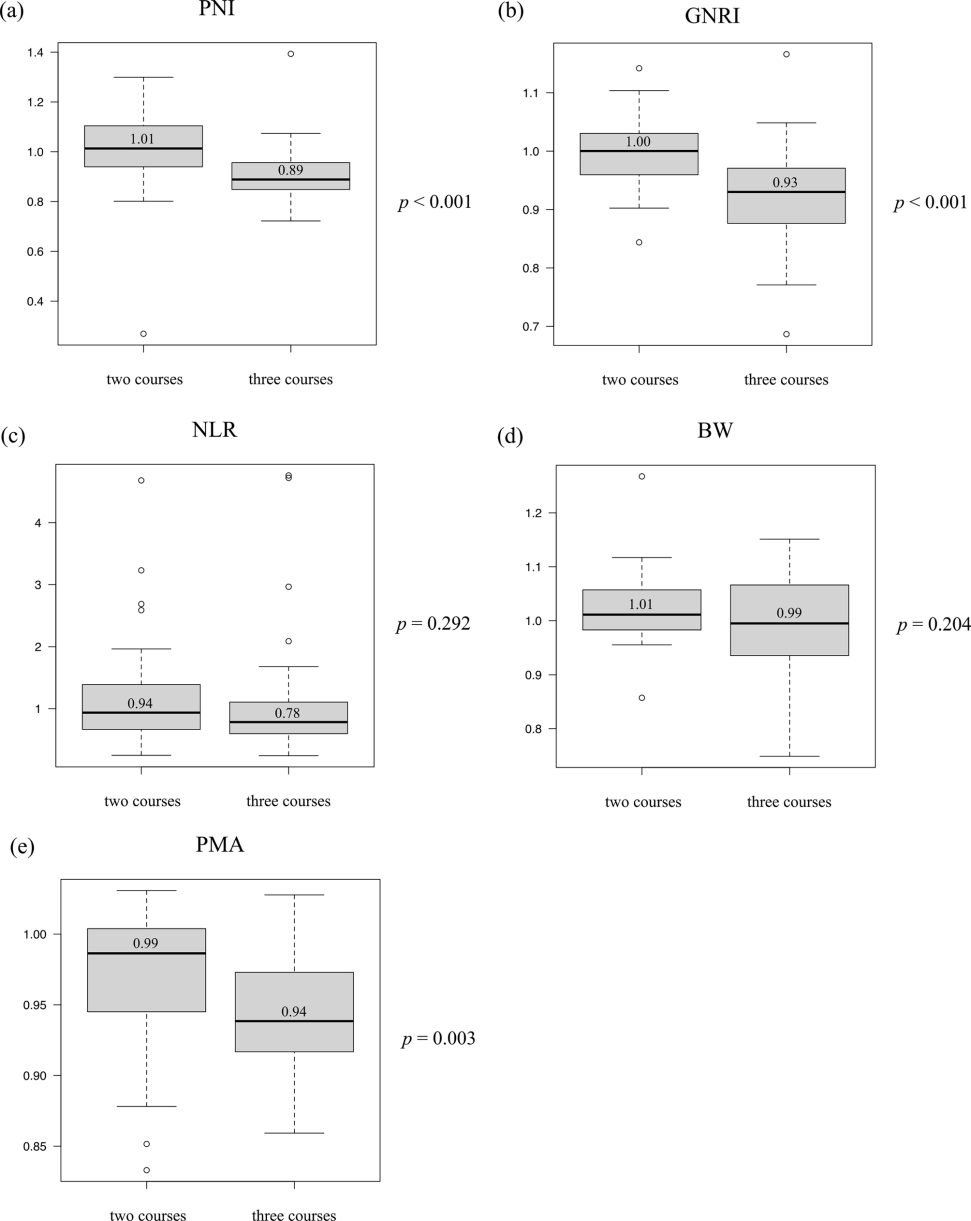
Changes in nutritional status and skeletal muscle volume before and after neoadjuvant chemotherapy (NAC). The change rates of multiple indicators associated with nutritional status and skeletal muscle volume between initial status and status after NAC were investigated. The change rates of the prognostic nutritional index (PNI) (**a**), geriatric nutritional risk index (GNRI) (**b**), neutrophil-to-lymphocyte ratio (NLR) (**c**), body weight (BW) (**d**), and psoas muscle area (PMA) (**e**) were compared between two and three courses of docetaxel, cisplatin, and 5-fluoroucaril (DCF) regimen. The comparisons of the change rates between two and three courses of DCF in PNI, GNRI, NLR, BW, and PMA were 1.01 (interquartile range [IQR]: 0.94–1.10) vs. 0.89 (IQR: 0.85–0.96); *p* < 0.001, 1.00 (IQR: 0.96–1.03) vs. 0.93 (IQR: 0.88–0.97); *p* < 0.001, 0.94 (IQR: 0.68–1.38) vs. 0.78 (IQR: 0.60–1.11); *p* = 0.292, 1.01 (IQR: 0.98–1.06) vs. 0.99 (IQR: 0.94–1.07); *p* = 0.204, and 0.99 (IQR: 0.95–1.00) vs. 0.94 (IQR: 0.92–0.97); *p* < 0.001, respectively

Figure 2 shows the comparisons of change rates of indicators related to nutritional status and skeletal muscle volume between the two- and three-course DCF groups. The PNI rate of change from the initial value to the preoperative value was significantly lower in the three-course group (1.01 vs. 0.89; *p* < 0.001) (Fig. 2a). Similarly, the GNRI rate of change was significantly lower in the three-course group (1.00 vs. 0.93; *p* < 0.001) (Fig. 2b). However, there were no significant differences in the NLR and body weight rates of change between the two groups (0.94 vs. 0.78; *p* = 0.292 and 1.01 vs. 0.99; *p* = 0.204) (Fig. 2c, d). The number of patients with increased GPS, including 0 to 1, 1 to 2, and 0 to 2, tended to be higher in the three-course group, but the difference was not significant (11 [18.3%] and 10 [24.4%] in the two- and three-course groups) (*p* = 0.467). Regarding skeletal muscle volume, the PMA change rate was significantly lower in the three-course group than the two-course group (0.99 vs. 0.94; *p* = 0.003) (Fig. 2e).

The factors affecting the differences in the NAC courses on the nutritional status and skeletal muscle volume in the elderly patients were similar to those of the all included patients (supplemental figure). Although PMA did not show the significant difference in PMA (*p* = 0.074), PNI and GNRI rates of change were significantly worse in the three-course group than in the two-course group (*p* = 0.020 and 0.014).

### Factors associated with the change in nutritional status and skeletal muscle volume during neoadjuvant treatment

According to the results of the comparisons of the rates of changes in nutritional status and skeletal muscle volume, the factors associated with the changes in PNI, GNRI, and PMA were investigated by performing univariate and multivariate analyses. In the univariate analysis for PNI change rate, an initial PNI < 45 and three courses of DCF were indicated as significantly associated factors. In the multivariate analysis, including these two factors as covariates, both factors were found to be factors independently associated with the PNI change (*B* = 0.129; *p* < 0.001 and *B* =  − 0.057; *p* = 0.022) (Table 3). In the univariate analysis for GNRI, a BMI ≥ 21, an initial PNI < 45, and three courses of DCF were significantly associated factors. In the multivariate analysis, all three factors were found to be independently associated factors (*B* =  − 0.033; *p* = 0.008, *B* = 0.062; *p* < 0.001, and *B* =  − 0.059; *p* =  − 0.084) (Table 4). In the univariate analysis for PMA change rate, three courses of DCF and cStage IV were significantly associated factors. In the multivariate analysis, both factors were found to be independently associated factors (*B* =  − 0.024; *p* = 0.011 and *B* =  − 0.025; *p* = 0.038) (Table 5).

**Table 3:** Uni- and multivariate analyses for the change rate of PNI between the initial and preoperative values

	Univariate analysis			Multivariate analysis		
	B	*p* value	95% CI	*B*	*p* value	95% CI
Age ≥ 70	0.008	0.762	− 0.047–0.064			
Male	− 0.064	0.185	− 0.158–0.031			
BMI ≥ 21	− 0.022	0.419	− 0.077–0.032			
Initial PNI < 45	0.146	< 0.001	0.097–0.194	0.129	< 0.001	0.080–0.179
Sarcopenia based on PMI	− 0.031	0.279	− 0.087–0.025			
Tumor location: Ut	0.015	0.706	− 0.066–0.097			
Three courses of DCF	− 0.092	< 0.001	− 0.144– − 0.040	− 0.057	0.022	− 0.105– − 0.009
Pathological response to NAC ≥ 1b	0.033	0.239	− 0.022–0.089			
cStage IV	− 0.044	0.205	− 0.113–0.025			

*PNI* prognostic nutritional index, *CI* confidence interval, *BMI* body mass index, *GNRI* geriatric nutritional risk index, *PMVI* psoas muscle index, *DCF* docetaxel + cisplatin + 5-fluorouracil, *NAC* neoadjuvant chemotherapy

**Table 4:** Uni- and multivariate analyses for the change rate of GNRI between the initial and preoperative values

	Univariate analysis			Multivariate analysis		
	*B*	*p* value	95% CI	*B*	*p* value	95% CI
Age ≥ 70	0.009	0.569	− 0.022–0.040			
Male	− 0.020	0.463	− 0.074–0.034			
BMI ≥ 21	− 0.050	0.001	− 0.079– − 0.021	− 0.033	0.008	− 0.058– − 0.009
Initial PNI < 45	0.090	< 0.001	0.064–0.117	0.062	< 0.001	0.036–0.088
Sarcopenia based on PMI	0.002	0.907	− 0.030–0.034			
Tumor location: Ut	− 0.013	0.577	− 0.059–0.033			
Three courses of DCF	− 0.075	< 0.001	− 0.102– − 0.047	− 0.059	< 0.001	− 0.084– − 0.035
Pathological response to NAC ≥ 1b	0.022	0.170	− 0.010–0.053			
cStage IV	− 0.025	0.215	− 0.064–0.014			

*GNRI* geriatric nutritional risk index, *CI* confidence interval, *BMI* body mass index, *PNI* prognostic nutritional index, PMI psoas muscle index, *DCF* docetaxel + cisplatin + 5-fluorouracil, *NAC* neoadjuvant chemotherapy

**Table 5:** Uni- and multivariate analyses for the change rate of PMA between the initial and preoperative values

	Univariate analysis			Multivariate analysis		
	*B*	*p* value	95% CI	*B*	*p* value	95% CI
Age ≥ 70	− 0.007	0.451	− 0.025–0.011			
Male	− 0.023	0.148	− 0.055–0.008			
BMI ≥ 21	− 0.007	0.477	− 0.025–0.012			
Initial PNI < 45	0.005	0.588	− 0.014–0.024			
Sarcopenia based on PMI	− 0.004	0.700	− 0.022–0.015			
Tumor location: Ut	0.006	0.667	− 0.021–0.033			
Three courses of DCF	− 0.031	0.001	− 0.049– − 0.014	− 0.024	0.011	− 0.042– − 0.006
Pathological response to NAC ≥ 1b	0.002	0.832	− 0.017–0.021			
cStage IV	− 0.036	0.002	− 0.058– − 0.014	− 0.025	0.038	− 0.048– − 0.001

*PMA* psoas muscle area, *CI* confidence interval, *BMI* body mass index, *PNI* prognostic nutritional index, *GNRI* geriatric nutritional risk index, *PMI* psoas muscle index, *DCF* docetaxel + cisplatin + 5-fluorouracil, *NAC* neoadjuvant chemotherapy

### Kaplan–Meier analyses of OS and RFS

**Fig. 3:**
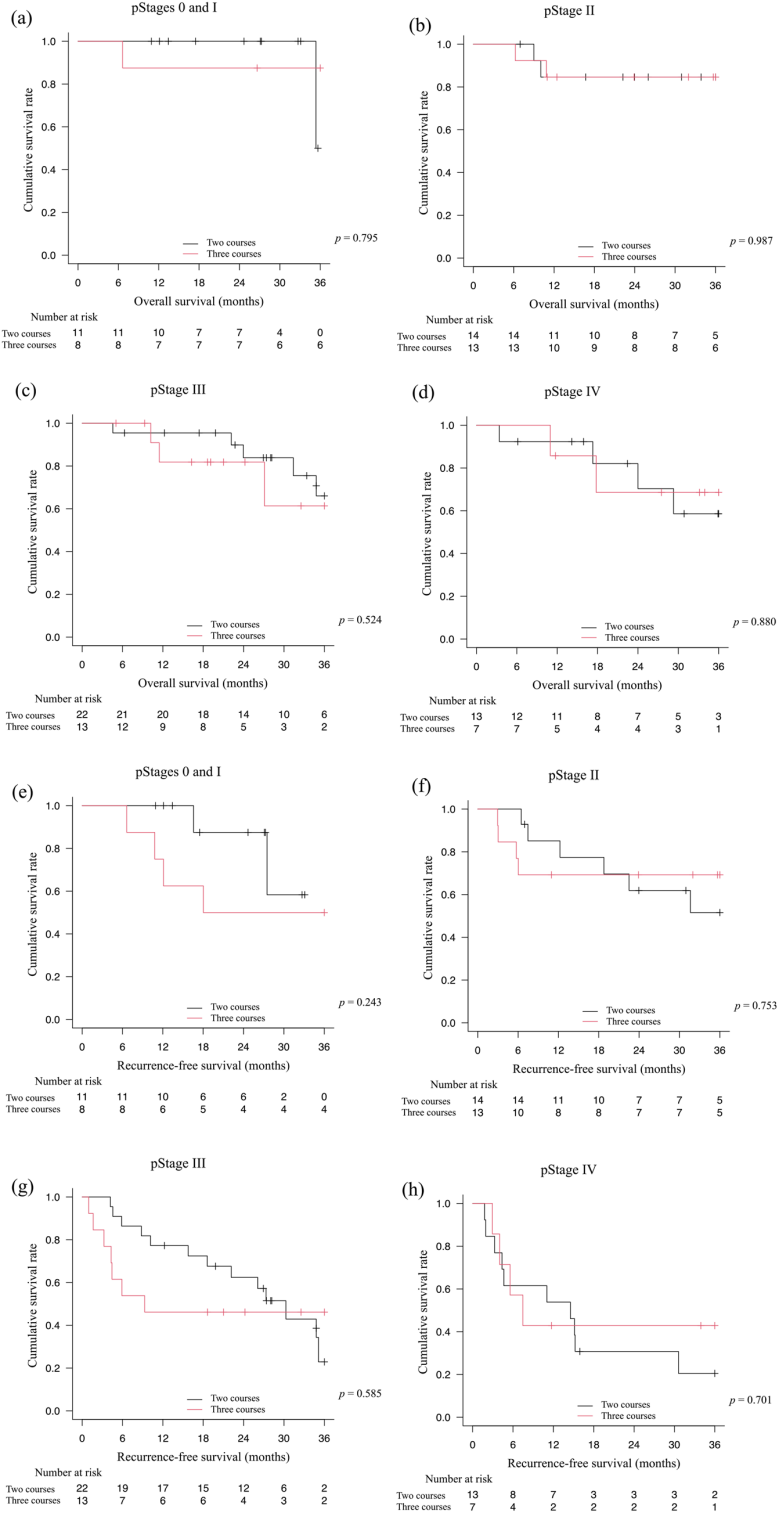
Comparison of long-term survival between the two- and three-course groups. Overall survival (OS) and recurrence-free survival (RFS) from Kaplan–Meier analyses were compared between the two- and three-course groups of neoadjuvant docetaxel, cisplatin, and 5-fluoroucaril (DCF) in the different pStages. The 3-year OS rates for the two- and three-course groups of DCF were 50.0% and 87.5% (*p* = 0.795) in pStages 0 and 1 (**a**), 84.6% and 84.6% (*p* = 0.987) in pStage II (**b**), 66.0% and 61.4% (*p* = 0.524) in pStage III (**c**), and 58.6% and 68.6% (*p* = 0.880) in pStage IV (**d**), respectively. The 3-year RFS rates for the two and three courses of DCF were 58.3% and 50.0% (*p* = 0.243) in pStages 0 and I (**e**), 51.6% and 69.2% (*p* = 0.753) in pStage II (**f**), 22.9% and 46.2% (*p* = 0.585) in pStage III (**g**), and 20.5% and 42.9% (*p* = 0.701) in pStage IV (**h**), respectively

The 3-year OS and RFS curves were compared between the two-course and three-course groups in the pStages I–IV (Fig. 3). None of the analyses in the different pStages showed statistically significant differences in OS and RFS between the two-course and three-course groups of neoadjuvant DCF.

## Discussion

As the intensity of the NAC for EC increased from doublet to triplet regimen, significantly higher incidences of severe adverse events were observed in DCF regimen than found with the former CF regimen [6]. Accordingly, the negative impact of DCF on preoperative nutritional status and skeletal muscle loss could be considered to a greater degree than observed with the former neoadjuvant CF. In addition, the Japanese EC practice guidelines also mention that administration of neoadjuvant DCF can be too intense in some patients, especially in those who are elderly and patients with various severe comorbidities [7]. A previous study also reported that DCF was superior to the CF regimen in long-term survival, while the survival benefit of DCF was not observed in patients ≥ 76 years [18]. In such situations, two courses of DCF, rather than the normal three courses, could become an additional clinical option. Moreover, Kubo et al. mentioned that continuing the third course of neoadjuvant DCF even had a risk of worsening survival in patients who did not experience a response to the prior two courses of DCF in locally advanced EC [19].

This study focused on the differences in the influences of the two and three courses of DCF on nutritional status and skeletal muscle change during neoadjuvant treatment. Our findings showed that two major nutrition-related indicators, PNI and GNRI, were significantly decreased during the NAC treatment in the three-course DCF and relatively maintained in the two-course DCF. Similarly, compared with two courses of DCF, three courses had a negative effect on the skeletal muscle volume during NAC. Furthermore, multivariate analysis showed that three courses of DCF was an independent risk factor for the rates of changes in PNI, GNRI, and PMA. On the contrary, there was no significant difference in NLR between the two groups, which might be because several patients experienced neutropenia as an adverse event of chemotherapy and required subsequent use of granulocyte colony stimulating factor. Similarly, no significant difference was observed in their GPS changes. This lack of an effect of the difference between two- and three-course DCF on the GPS changes could be because the crude cutoff setting that was designed for a screening method made it difficult to reveal the difference. When comparing hematological adverse events, neutropenia with CTCAE grade ≥ 2 was significantly higher in the three-course than the two-course group. The survival analysis showed no significant difference between the two-course and three-course groups in any pStage. Although it was difficult to completely avoid bias related to the differences in the patients’ backgrounds between the two groups, our findings indicated that the three-course DCF regimen was inferior to the two-course regimen, especially with respect to the nutritional status and skeletal muscle loss.

Our univariate and multivariate analyses for multiple indicators showed other interesting findings. Although three courses of DCF had negative effects on PNI and GNRI, low nutritional status with a PNI < 45 at the initial diagnosis was positively associated with the change rates of PNI and GNRI. These findings indicated that patients with good nutritional condition can be more susceptible to NAC than those who are persistently malnourished due to EC conditions. We usually consider providing nutritional support for patients who are malnourished, however, even the patients in good nutritional condition can have a risk of nutritional disorder when receiving intense neoadjuvant treatment.

Several previous studies have reported the clinical importance of nutritional support during neoadjuvant treatment [20, 21], and multiple previous studies have shown that the skeletal muscle loss even during NAC was associated with postoperative complications and reduced long-term survival [22–24]. EC is prone to causing nutritional disorders due to the tumor’s location and requires a long treatment period from the initial diagnosis to radical esophagectomy that is highly invasive; therefore, treatment strategies that can maintain nutritional status and skeletal muscle volume are essential. Our results showed that two courses of DCF should be considered as a treatment option.

We should note that our results do not support recommending two courses of neoadjuvant DCF in all patients. The strongest evidence of the superiority of DCF to CF in NAC for locally advanced EC was primarily based on the JCOG 1109 study with three courses of DCF. Regarding tumor shrinkage achieved by radical surgery and reduction of postoperative recurrence due to control of micrometastases, three courses of DCF was considered to have a greater clinical benefit than two courses, except for DCF-resistant EC. In clinical practice, the number of administered courses should be comprehensively determined according to the clinical situation, including the patient’s overall condition and degree of cancer progression; however, when considering the highly negative effect of three courses on nutritional status and skeletal muscle loss, more aggressive intervention to prevent malnutrition and sarcopenia during neoadjuvant treatment is essential when three courses of DCF are administered as NAC.

This study had several limitations. First, the biases due to the retrospective study design, especially in the patients’ characteristics, could not be avoided. Actually, although administration of two or three courses was only related to whether or not the treatment was given in the early or later study period, some patients with highly advanced EC, such as Stage IV EC, received three courses even in the later study period. This bias probably influenced the patient characteristics, particularly the cancer stage. To minimize the influence of these limitations, this study reviewed all patients who underwent esophagectomy after neoadjuvant DCF during the study period to evaluate the factors that had effects on reduced nutritional status and greater skeletal muscle loss by performing multivariate analysis. Moreover, in the analysis of long-term survival, the analysis was stratified by pStage. Second, nutritional status and skeletal muscle volume were evaluated only from blood-test data and computed tomography images, respectively, in this study. Malnutrition and skeletal muscle loss resulting in sarcopenia should be assessed by multiple measures, including body composition, physical assessment (such as muscle strength and gait speed), history of oral intake, and daily activity status; therefore, detailed assessment of the influence of NAC on sarcopenia was difficult. However, these indicators, including PNI, GNRI, and PMA, are useful and typical measures that are easy to measure in clinical settings. It is easy to confirm reproducibility, and further clinical research and applications are expected in the future. Third, the risk of selection bias as this was single-center study could not be excluded. The study sample size was not large, and the management of NAC for EC probably varies among institutions; therefore, generalizing from our comparison of two-course and three-course DCF based only on our findings is probably unwarranted without additional supportive studies. Despite these limitations, we believe that our findings provide important information from a new perspective related to novel NAC using DCF regimen.

In conclusion, this study showed that worsening of nutritional status and greater skeletal muscle loss during NAC were more serious with three courses than with two courses of neoadjuvant DCF. If only considering the effects of nutritional status and skeletal muscle volume, our results showed that two courses were superior to three courses of neoadjuvant DCF.

## Supplementary Information

Below is the link to the electronic supplementary material.

Supplementary file1 (PDF 481 KB)

## Data Availability

Due to the nature of this research, the study participants did not agree the use of their data publicly; thus, supporting data is unavailable.
