# Supplementary material for: Clinical advantages of two vs. three courses of neoadjuvant chemotherapy using docetaxel + cisplatin + 5-fluorouracil to improve preoperative nutritional status and mitigate decreasing skeletal muscle in resectable esophageal cancer
Source: Int J Clin Oncol. 2025 Jul 17;30(10):1992–2002. doi: 10.1007/s10147-025-02839-6 (PMC12474620; doi:10.1007/s10147-025-02839-6)

Supplemental Figure. Changes in nutritional status and skeletal muscle volume before and after neoadjuvant chemotherapy (NAC) in the elderly patients

The change rates of the prognostic nutritional index (PNI) (a), geriatric nutritional risk index (GNRI) (b), neutrophil-to-lymphocyte ratio (NLR) (c), body weight (BW), and psoas muscle area (PMA) (e) were compared between two and three courses of a docetaxel, cisplatin, and 5-fluoroucaril (DCF) regimen in the patients aged ≥ 75 years old. The change rates for two and three courses of DCF in PNI, GNRI, NLR, BW, and PMA were 1.01 (interquartile range [IQR]: 0.94–1.04) vs. 0.92 (IQR: 0.87–0.94); $p = 0.020$, 0.99 (IQR: 0.96–1.02) vs. 0.93 (IQR: 0.89–0.95); $p = 0.014$, 0.99 (IQR: 0.71–1.29) vs. 0.60 (IQR: 0.52–0.94); $p = 0.095$, 0.98 (IQR: 0.97–1.01) vs. 0.98 (IQR: 0.91–0.99); $p = 0.452$, and 0.99 (IQR: 0.96–1.00) vs. 0.93 (IQR: 0.92–0.96); $p = 0.074$, respectively.

(a)

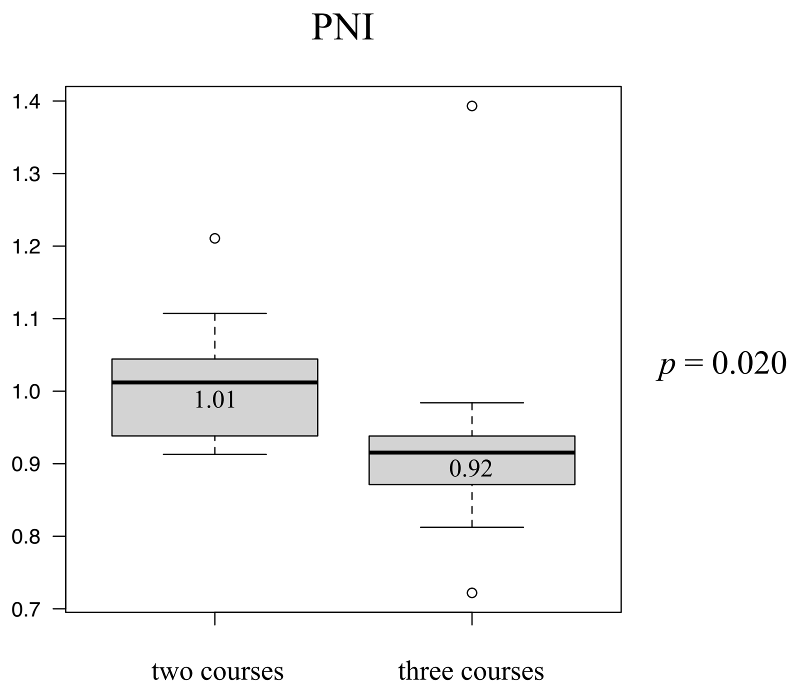
PNI
1.4
1.3
1.2
1.1
1.0
0.9
0.8
0.7
1.01
0.92
two courses
three courses
$p = 0.020$

(b)

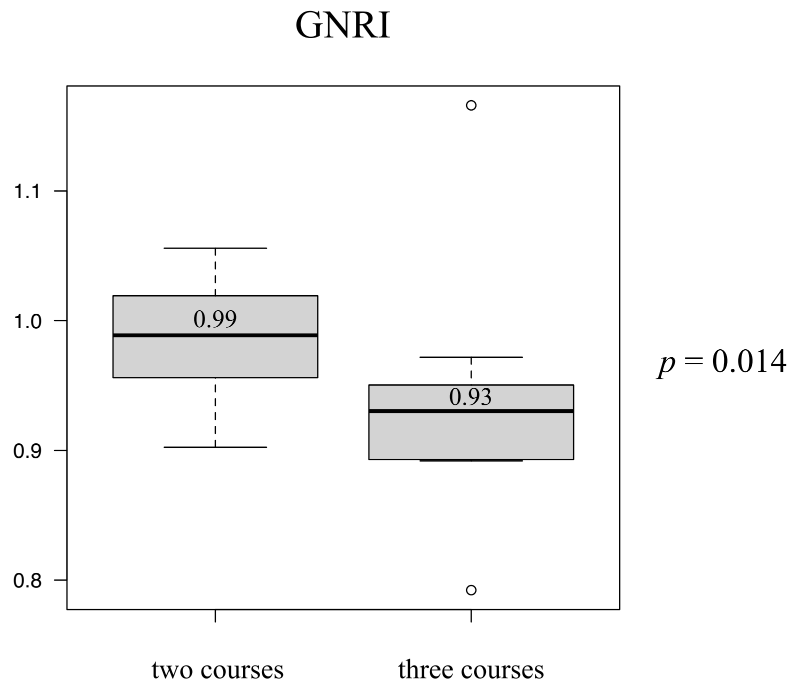
GNRI
1.1
1.0
0.9
0.8
0.99
0.93
two courses
three courses
$p = 0.014$

(c)

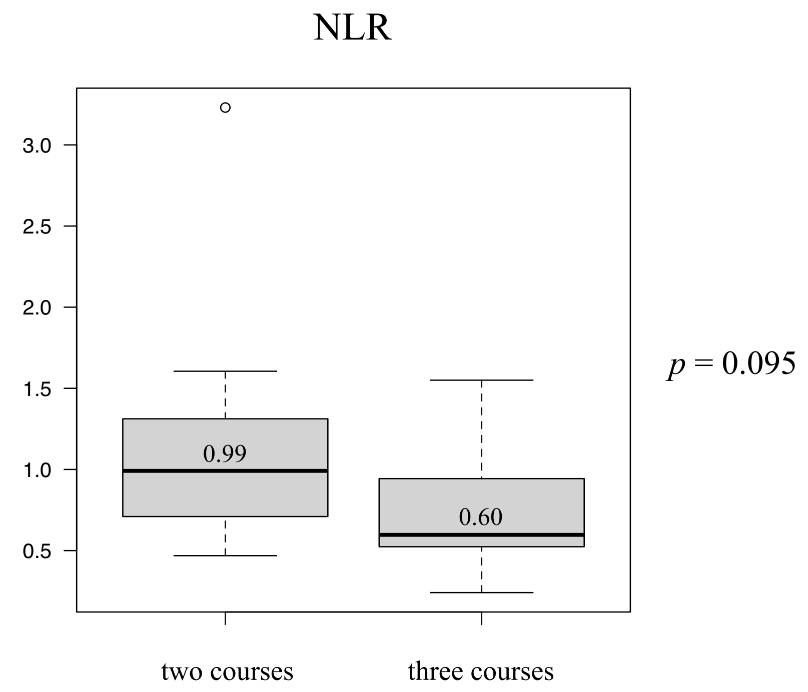
NLR
3.0
2.5
2.0
1.5
1.0
0.5
0.99
0.60
two courses
three courses
p = 0.095

(d)

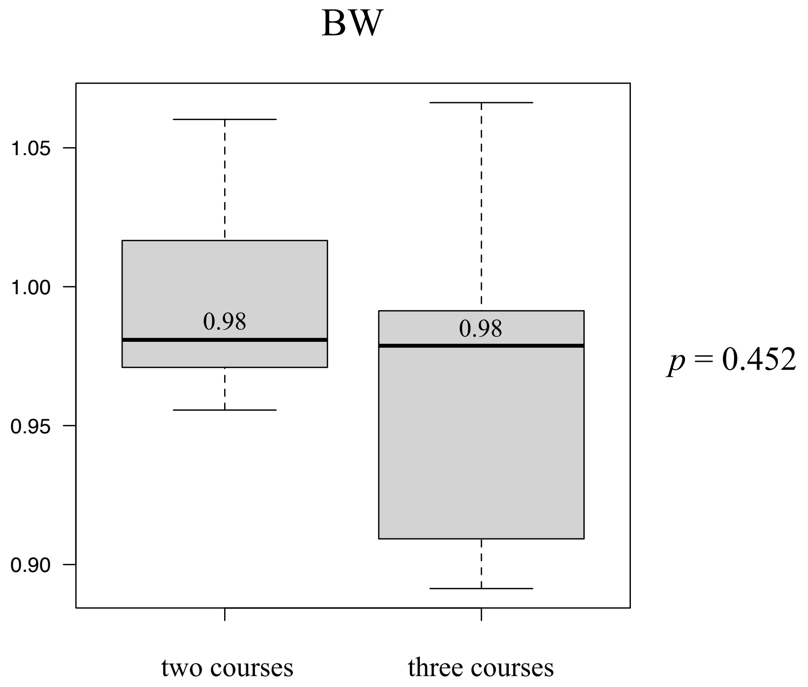
BW
1.05
1.00
0.95
0.90
0.98
0.98
two courses
three courses
p = 0.452

(e)

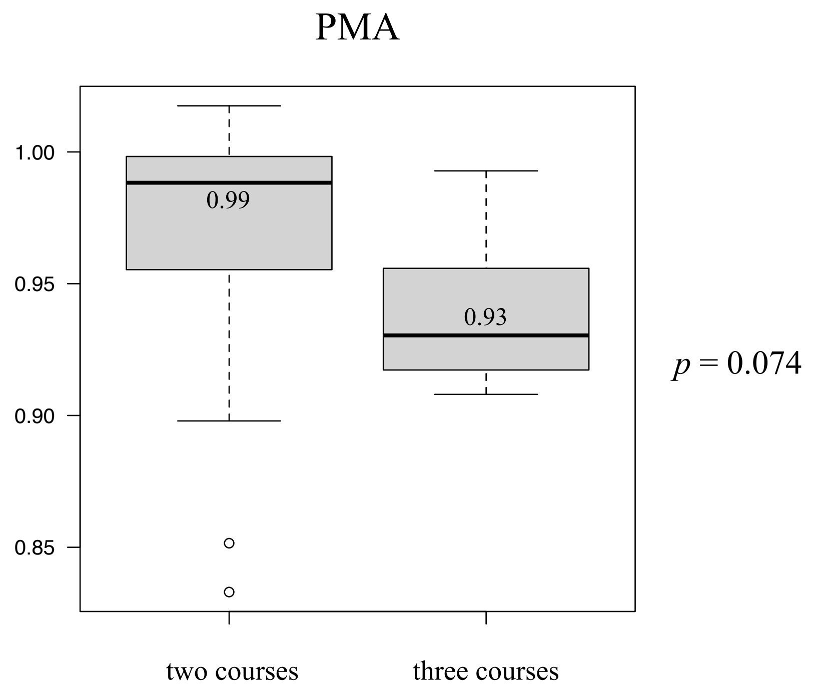

Supplement: Supplementary file 1 — Supplementary file1 (PDF 481 KB) [file 10147_2025_2839_MOESM1_ESM.pdf]
